# Characterization of aggregate/aggresome structures formed by polyhedrin of *Bombyx mori* nucleopolyhedrovirus

**DOI:** 10.1038/srep14601

**Published:** 2015-10-06

**Authors:** Zhong-Jian Guo, Liu-Xing Tao, Xian-Yun Dong, Meng-Han Yu, Ting Tian, Xu-Dong Tang

**Affiliations:** 1 Institute of Life Sciences, Jiangsu University, 301# Xuefu Road, Zhenjiang 212013, Jiangsu, P.R. China; 2 College of Biotechnology, Jiangsu University of Science and Technology, 2# Mengxi Road, Zhenjiang 212018, Jiangsu, P.R. China

## Abstract

Virus infections often lead to formation of aggregates and aggresomes in host cells. In this study, production of aggregates and aggresomes by the highly expressed protein polyhedrin of *Bombyx mori* nucleopolyhedrovirus (BmNPV) at 24 h postinfection (p.i.) was detected with a fluorescent molecular dye, and verified by colocalization of polyhedrin with aggresomal markers, GFP-250 and γ−tubulin. Polyhedrin aggregates showed hallmark characteristics of aggresomes: formation was microtubule-dependent; they colocalized with heat shock cognates/proteins of the 70-kDa family (HSC/HSP70s), ubiquitinated proteins and recruited the mitochondria. Aggregated polyhedrin protein gradually gained its active conformation accompanying progress of BmNPV infection. At 48 h p.i. recovered polyhedrin bound directly to *Bombyx mori* microtubule-associated protein 1-light chain 3 (BmLC3), an autophagosome marker, and was colocalized with BmLC3 to the isolation membrane of autophagosome, implying the involvement of polyhedrin in cellular autophagy. Inhibition of autophagy by 3-methyladenine (3-MA) dramatically resulted in decrease of polyhedrin expression and polyhedra particle production. These observations suggested that highly expressed polyhedrin forms aggregate to get involved in cellular autophagy then play an important role in polyhedra production.

A hallmark of many neurodegenerative disorders, such as Alzheimer’s disease, Huntington’s disease, Parkinson’s disease and oculopharyngeal muscular dystrophy, is the presence of misfolded protein aggregates^1^. In eukaryotic cells, aggregates may form because of genetic mutation, errors in transcription, mRNA processing or translation. Alternatively, they can be produced in response to some environmental factors, such as hyperthermia, exposure to reactive oxygen species and chemical treatment^2^,^3^. Once protein aggregates have formed, they tend to pose a substantial burden to protein homeostasis in cells. Therefore, cells remove toxic protein aggregates using a variety of homestatic mechanisms, such as refolding by some protein chaperones and remodeling factors to obtain their active conformations^4^, and degradation by the ubiquitin-proteasome pathway and chaperone-mediated autophagy^5^. When the cellular degradative capacity is exceeded, aggregates are delivered to the microtubule organizing center (MTOC) by dynein-dependent retrograde transport along microtubules to perinuclear sites of aggregate depositions referred to as aggresomes which may serve as storage depots allowing degradation by autophagy^6^.

Increasing evidences suggest that aggregates and aggresomes, which differ by their location, size, content and putative function, form in response to viral infection. For instance, in adenovirus-infected cells, expression of either E4 11k or E1b 55k, can individually induce aggresome formation, and both relocate Mre11-Rad50-Nbs1 complex^7^,^8^,^9^, and E4 11k protein also relocalizes the cytoplasmic P-body component Ddx6 to aggresomes^10^. Infection of herpes simplex virus led to the formation of aggresomes and some tegument proteins such as UL46 and VP16 were targeted to aggresomes^11^,^12^.

Baculoviruses are a family of DNA viruses that have a large, circular, supercoiled and double-stranded DNA genome within a rod-shaped nucleocapsid^13^. It was reported that baculovirus *Autographa californica* multiple nucleopolyhedrovirus (AcMNPV) produced aggresomes, resulting in proteotoxicity in Sf9 cells^14^,^15^. However, aggregates and aggresomes formed during infection of baculovirus have rarely been characterized. Polyhedrin, the highly expressed protein in life cycle of nucleopolyhedrovirus, is generally considered as a structural component to stabilize baculovirus virions in the environment allowing them to persist indefinitely^16^. Nevertheless, the role of polyhedrin, rather than as a protective structural component of polyhedra, has not been observed. In this study aggregates and aggresomes produced by polyhedrin were determined. These cytoplasmic foci displayed hallmark characteristics of aggresomes: microtubule-dependent formation, colocalization with HSC/HSP70s and ubiquitinated proteins, recruitment of the mitochondria. At 48 h p.i., conformation-recovered polyhedrin interacted with BmLC3 and colocalized with BmLC3 to the isolation membrane of autophagosome, implying the involvement of polyhedrin in cellular autophagy. The autophagy played an important role in polyhedrin expression and polyhedra particle production, as evidenced by the findings that inhibition of autophagy led to decrease of polyhedrin expression and polyhedra production.

## Results

### Detection of protein aggregates/aggresomes during BmNPV infection

**Figure 1:**
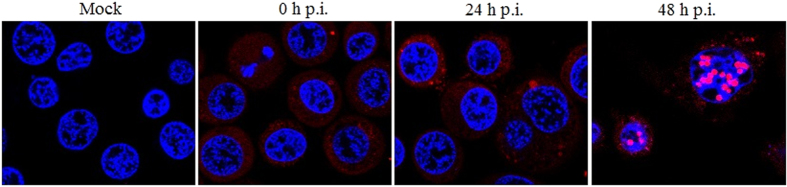
Protein aggregation detected by ProteoStat^®^ dye. BmN cells were either mock- or infected with BmNPV BVs at an MOI of 10 TCID_50_/cell. After treatment, cells were incubated with ProteoStat^®^ dye for 30 min. Cell nuclei were stained with Hoechst 33342. Images were obtained using a Leica TCS SP5 confocal laser-scanning microscope.

ProteoStat Aggresome Detection Kit has been used extensively to specifically detect denatured and/or misfolded protein aggregates and inclusion bodies in *Escherichia coli*, plant and mammalian cells^17^,^18^,^19^,^20^,^21^,^22^,^23^. During BmNPV infection, protein aggregates/aggresomes (foci) were detected by this dye to be localized largely in the cytoplasm. Some dots that represents polyhedral occlusion bodies (OBs), were also observed in the nucleus at 48 h p.i. (Fig. 1).

### Colocalization of viral protein polyhedrin with aggregates/aggresomes

**Figure 2:**
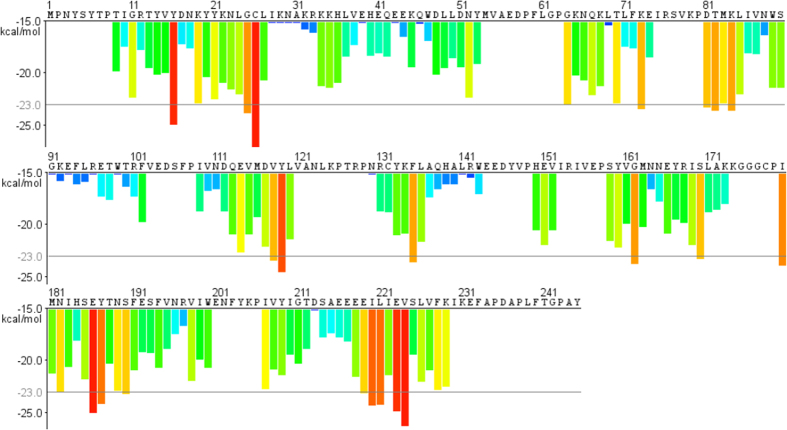
Aggregation-prone stretches of polyhedrin predicted using the 3D profile method. Protein sequence was submitted online http://services.mbi.ucla.edu/zipperdb. The amino acid residues were colored according to their fibrillation propensities. Stretches with energy below a value of –23 kcal/mol showed high fibrillation propensities.

**Figure 3:**
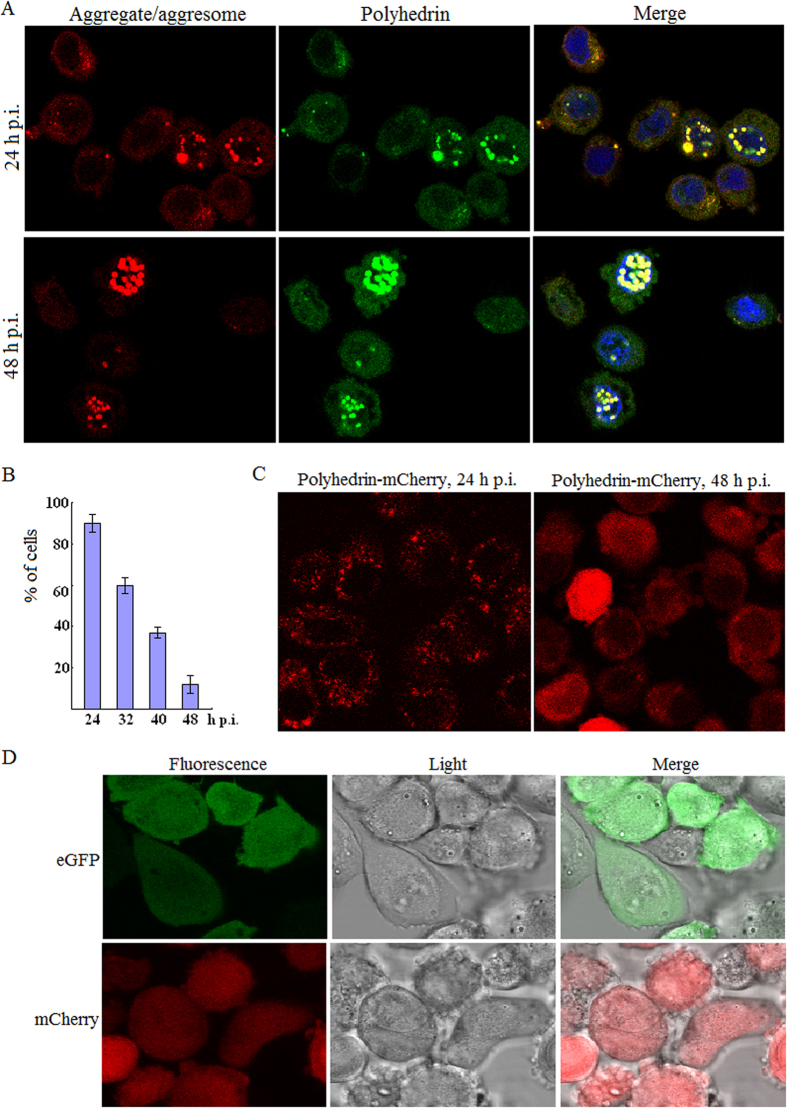
Polyhedrin, the protein expressed in a large amount, formed aggregates/aggresomes. (**A**) Colocalization between polyhedrin and aggregates/aggresomes in the cytoplasm. Cells were infected with BmNPV T3 isolate at an MOI of 10 TCID_50_/cell, and at selected time points fixed and permeabilized, then incubated in the blocking buffer, followed by incubation with mouse monoclonal anti-polyhedrin antibody and FITC-conjugated goat anti-mouse IgG. Next, cells were stained with ProteoStat^®^ dye and Hoechst 33342 prepared according to kit instructions for 30 min at room temperature, washed with 1 × PBS and then imaged by a fluorescence microscopy using a Texas Red filter set for the ProteoStat^®^ dye, and an FITC filter set for FITC-conjugated antibody respectively. (**B**) Percentage of cells displaying colocalization between aggregates/aggresomes and polyhedrin in the cytoplasm. Each percentage value is obtained from three independent infections, and for each infection, 90–120 cells were counted. Error bars indicates ±SEM. (**C**) Fusion protein polyhedrin-mCherry aggregated (foci) in the cytoplasm at 24 h p.i. Polyhedrin-mCherry was expressed using the BmNPV Bac-to-Bac system then visualized under a confocal laser scanning microscope at 24 and 48 h p.i. (**D**) Fluorescent proteins eGFP and mCherry alone do not localize to cytoplasmic foci. Plasmids pP_ie1_-eGFP, pP_ph_-eGFP and pP_ph_-mCherry (Supplementary Table S2) were transposed. Positive bacmids were transfected into BmN cells to obtain BV which was used for infection. Fluorescent cells were visualized at 24 or 48 h p.i.

As described above, infection of BmNPV could induce formation of aggregates and aggresomes, raising the question of where these toxic structures come from. During BmNPV progeny production, a large number of viral proteins, e.g. polyhedrin, are synthesized in a relatively short time and a large amount, whereby protein folding can become a limiting step for their active conformation and trafficking. It was inferred that some proteins encoded by BmNPV genome formed aggregates and aggresomes. Firstly, to address it, the fibrillation propensity profiles within polyhedrin amino acid sequence were assessed by a 3D profile method. Energy per amino acid below a threshold value of –23 kcal/mol was indicative of a high fibrillation propensity. Segments obtained from 3D profile method were showed in the image where the aggregation-prone regions were found to be Gly^25^-Cys^26^, Asp^81^-Lys^84^, Glu^186^-Tyr^187^ and Glu^219^-Val^224^ (Fig. 2). Then co-staining of aggregates/aggresomes for polyhedrin was performed. BmN cells were infected, fixed and permeabilized at 24 h p.i., and then stained with a mouse monoclonal anti-polyhedrin antibody and the ProteoStat^®^ dye. As shown in Fig. 3A (upper panel), colocalization between aggregates/aggresomes and polyhedrin was evident at 24 h p.i.

A time course analysis of colocalization between aggregates/aggresomes and polyhedrin was performed, and the percentage of cells displaying colocalization in each infection was calculated. At 24 h p.i., a higher percentage, approximately 90%, of the infected cells showed co-staining for polyhedrin and aggregates/aggresomes (Fig. 3A,B). A steady decrease in the number of cells displaying colocalization in the cytoplasm was observed accompanying the progress of BmNPV infection, as demonstrated by the percentages of cells exhibiting co-staining were about 60% at 32 h p.i., 37% at 40 h p.i. and 12% at 48 h p.i. (Fig. 3B), suggesting aggregated polyhedrin may be folded and/or removed.

Some other viral proteins such as products of open reading frame 75 and 101 were also aggregated, which could be detected by ProteoStat^®^ dye (Our unpublished data). To confirm further these results that polyhedrin was specifically aggregated, the protein polyhedrin fused to N-terminal of mCherry was expressed using BmNPV bacmid system, and then viewed under a confocal laser scanning microscope. At 24 h p.i. polyhedrin-mCherry was localized mainly in the cytoplasm, displaying foci, whereas at 48 h p.i. in both the cytoplasm and the nucleus, exhibiting a more diffuse pattern (Fig. 3C). To determine that overexpression of fluorescent proteins does not result in aggregate/aggresome formation, localization of monomeric eGFP and mCherry alone, driven by *ie1* or *polyhedrin* promoter, was tested. No cytoplasmic foci were found for fluorescent proteins eGFP and mCherry that were distributed diffusely throughout the whole cell (Fig. 3D). These findings indicated that viral polyhedrin formed aggregates/aggresomes.

### Polyhedrin was targeted to aggresomes

**Figure 4:**
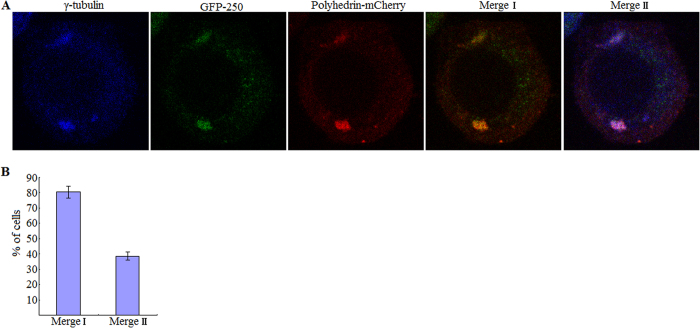
The polyhedrin was targeted to aggresome. (**A**) Colocalization of polyhedrin-mCherry and the heterogenous aggresomal marker GFP-250, and of coalesced aggregates of both GFP-250 and polyhedrin-mCherry with γ−tubulin. Plasmids pP_ie1_-GFP-250 and pP_ph_-Polyhedrin-mCherry were employed for transposition and transfection. Equal MOI (10 TCID_50_/cell) of P2 viral stocks individually expressing GFP-250 and polyhedrin-mCherry were used to co-infect BmN cells and then analyzed at 24 h p.i. by a confocal fluorescence microscopy, and in parallel, by immunostaining for γ−tubulin. In merged images, yellow dots suggested colocalization between GFP-250 and polyhedrin-mCherry (Merge I), and white ones indicated colocalization of coalesced GFP-250/polyhedrin-mCherry aggregate with γ−tubulin (Merge II). (**B**) Percentage of cells showing colocalization of GFP-250 with polyhedrin-mCherry (Merge I), and of coalesced GFP-250/polyhedrin-mCherry aggregate with γ−tubulin (Merge II). Each value is summarized from three independent infections. For each infection, 174–190 cells were scored for complete and partial colocalization data collection. Error bars indicates ±SEM.

As an efficient way for cell to avoid proteotoxicity, aggresome is formed to sequester and inactivate these potentially harmful aggregated proteins. These findings above raise the possibility that cytoplasmic foci could represent targeting of polyhedrin proteins to aggresomes. Thus to confirm whether or not some polyhedrin aggregates are aggresomes, the heterogenous aggresomal marker GFP-250, together with the fusion protein polyhedrin-mCherry, was expressed in BmN cells. Aggresomes formed by polyhedrin were portrayed by colocalization of chimera GFP-250 with polyhedrin-mCherry. GFP-250 consists of eGFP fused at its C-terminus to the first 250 amino acids of p115, and was shown to result in a spherical aggresome in previous study^24^. At 24 h p.i., co-infected cells contained aggregates of both GFP-250 and polyhedrin-mCherry in the cytoplasm. Moreover, there was a high degree of colocalization between these aggregates, as evidenced by complete and partial colocalization of GFP-250 with polyhedrin-mCherry in 80.5 ± 3.9% of BmN cells (Fig. 4A,B).

A *bona fide* aggresome is characterized by juxtanuclear location and colocalization with centrosome markers including γ−tubulin^2^,^7^,^10^. To determine whether these coalesced aggregates of both GFP-250 and polyhedrin-mCherry colocalized with γ−tubulin, BmN cells were co-infected with two recombinant viruses individually expressing GFP-250 and polyhedrin-mCherry, then fixed, permeabilized and stained for γ−tubulin at 24 h p.i. using a rabbit polyclonal anti-γ−tubulin antibody^25^,^26^. Nearly 38.5 ± 2.7% of cells contained aggregated GFP-250/polyhedrin-mCherry foci were co-stained for γ-tubulin at perinuclear locations, confirming that they were *bona fide* aggresomes. These aggresomes presented two structure types, the single sphere and extended ribbon (Fig. 4A,B). These data strongly indicated that a subpopulation of GFP-250 and polyhedrin-mCherry aggregated in cells were targeted to the same subcellular aggresomes.

### Polyhedrin foci possess other hallmark characteristics of aggresomes

**Figure 5:**
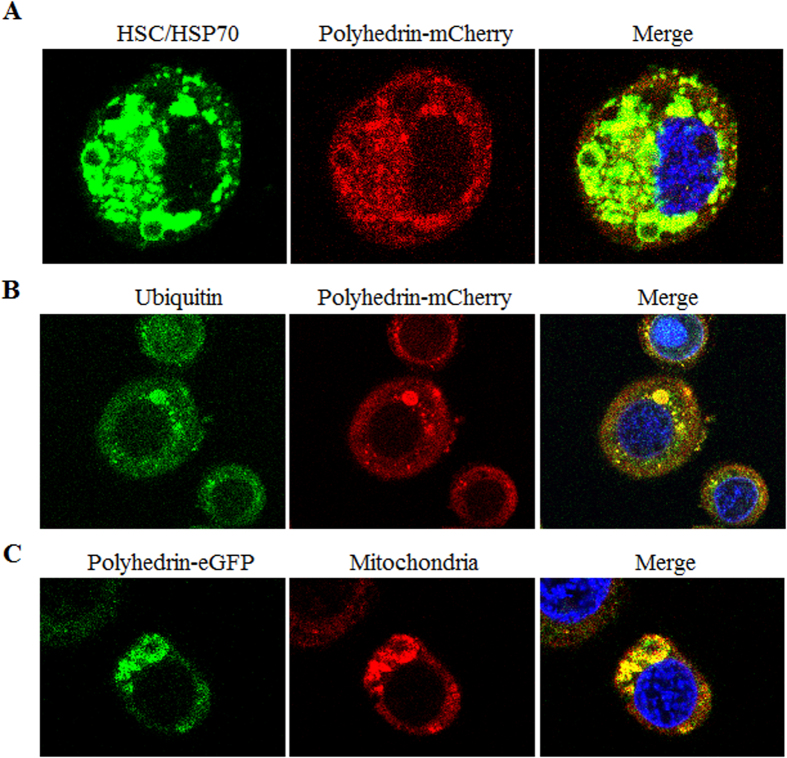
Colocalization analyses of aggregated polyhedrin with HSC/HSP70s, ubiquitinated proteins and mitochondria. Infected cells expressing polyhedrin-mCherry and polyhedrin-eGFP were collected at 24 h p.i., treated with paraformaldehyde and Triton X-100, and then immunostained for HSC/HSP70s (**A**) and ubiquitin (**B**). The mitochondria (**C**) were stained with a MitoTracker^®^ deep red probe (Molecular Probes, Life technologies, Eugene, OR, USA). Cell nuclei were stained with Hoechst 33342. Cells were imaged under a Leica TCS SP5 confocal laser-scanning microscope.

Next, whether polyhedrin foci possess other hallmark characteristics of aggresomes was demonstrated. Formation of aggresomes has been reported to recruit cytosolic components such as chaperones, ubiquitinated proteins and mitochondria, to facilitate clearance of aggregated proteins^27^,^28^. To characterize further that polyhedrin aggregates are aggresomes, association of HSC/HSP70, ubiquitin and mitochondria with polyhedrin was investigated. BmN cells were infected with the virus to highly express fusion protein polyhedrin-mCherry. After treatment by paraformaldehyde and Triton X-100, cells were immunostained for HSC/HSP70 and ubiquitinated proteins. Clearly, a subpopulation of HSC/HSP70s and ubiquitinated proteins colocalized with polyhedrin-mCherry in the cytoplasm to aggregated foci (Fig. 5A,B). Aggregates caused proteotoxicity in cells and they often recruit mitochondria to provide ATP required by chaperones and proteasomes for aggregated protein refolding and/or degradation. To investigate colocalization of mitochondria with polyhedrin aggresome structures, BmN cells were infected with a virus to highly express the fusion protein polyhedrin-eGFP, and then assessed by staining cells with a mitochondrion-selective dye, MitoTracker Red. Results showed these polyhedrin-eGFP foci colocalized with mitochondria (Fig. 5C). These data therefore provide additional evidences that some foci formed by polyhedrin are aggresomes.

### Formation of polyhedrin foci is microtubule-dependent

**Figure 6:**
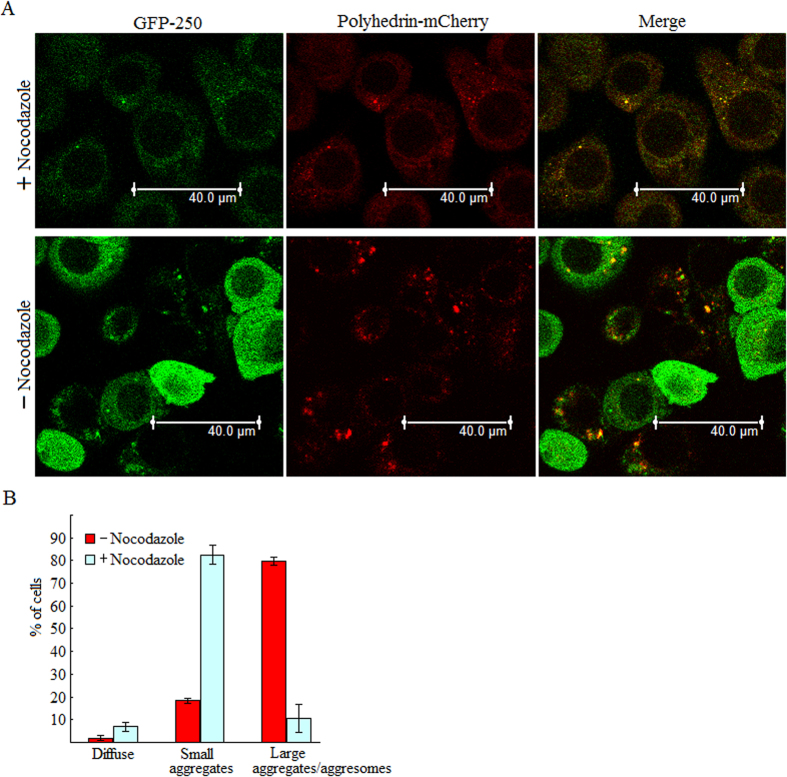
Formation of polyhedrin foci is microtubule-dependent. (**A**) Microtubule disruption prevents the formation of coalesced polyhedrin aggregates. BmN cells were co-infected with viral stocks individually expressing GFP-250 and polyhedrin-mCherry, treated with nocodazole at 0 h p.i., and then imaged by a confocal fluorescence microscopy at 24 h p.i. (**B**) Bar graph describing the effect of nocodazole on polyhedrin aggregate size. Each value is summarized from three independent infections. For each infection, 90–143 cells were scored. Error bars indicates ±SEM.

Aggresome formation occurs when small aggregates of misfolded and/or unfolded proteins are delivered towards the MTOC in a minus-end direction along the microtubule tracks; therefore microtubule disruption by drug nocodazole will prevent formation of a *bona fide* aggresome. To determine whether microtubule disruption would prevent the formation of polyhedrin aggresomes, co-infected cells expressing GFP-250 and polyhedrin-mCherry were treated with nocodazole at 0 h p.i., and then viewed at 24 h p.i. by a confocal microscope. Polyhedrin-mCherry displayed diffuse pattern, small and large aggregate distribution in the cytoplasm (Fig. 6A). About 20.3 ± 1.8% of untreated cells and upon treatment with nocodazole approximately 89.4 ± 6.0% of cells displayed diffuse pattern and small aggregate distribution of polyhedrin-mCherry. There was a significant shift from 79.9 ± 1.8% of untreated to 10.6 ± 6.0% of nocodazole-treated polyhedrin-mCherry-expressing cells that contained large aggregates (Fig. 6B). These observations suggested that formation of polyhedrin aggregates is microtubule-dependent.

### Polyhedrin interacts directly with the autophagosome marker, BmLC3

**Figure 7:**
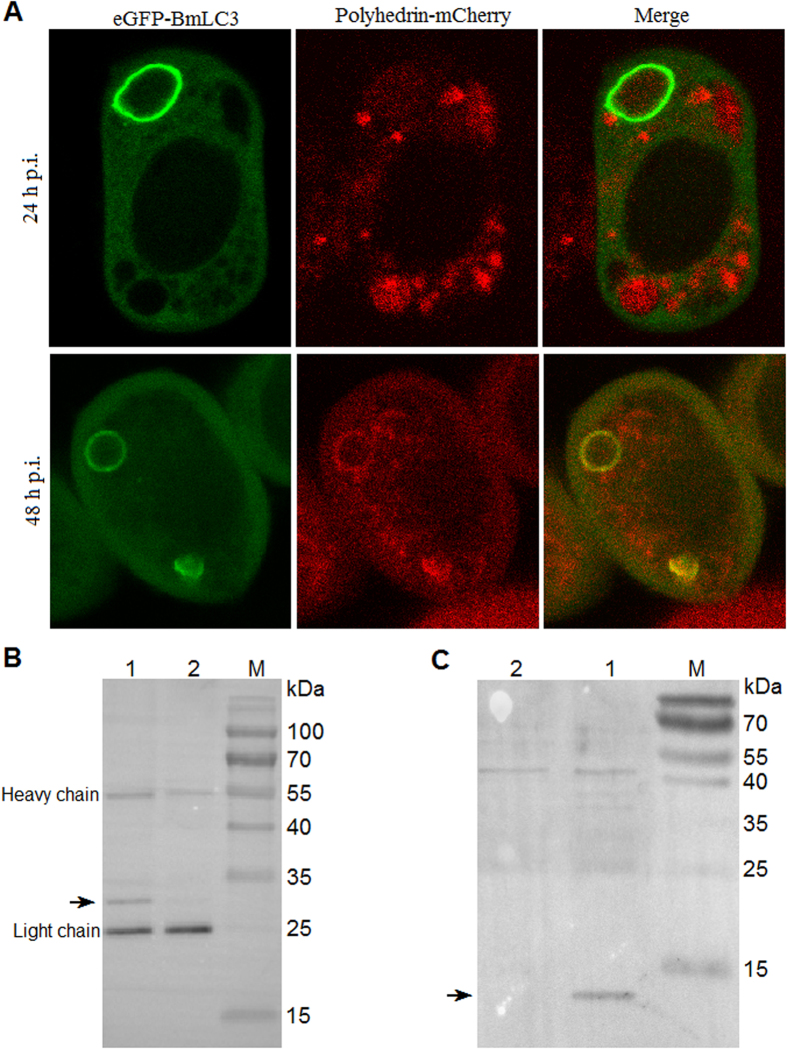
Polyhedrin interacts and is colocalized with BmLC3 on the isolation membrane of autophagosome. (**A**) Subcellular localizations of polyhedrin-mCherry and eGFP-BmLC3. The spherical structures indicated autophagosomes. Polyhedrin-mCherry was not at 24 h p.i. (upper panel) but at 48 h p.i. (lower panel) colocalized with BmLC3 on the isolation membrane of autophagosome. (**B**) eGFP-BmLC3 co-immunoprecipitates with polyhedrin. Plasmids pP_ie1_-eGFP-BmLC3 and pP_ie1_-eGFP were applied for transposition and transfection. BmN cells were co-infected with equal MOI (10 TCID_50_/cell) of a P2 viral stock and the BmNPV T3 isolate. eGFP or eGFP fused BmLC3 were immunoprecipitated from total cellular extract with a μMACS GFP Isolation Kit and subjected to SDS-PAGE. Co-purified polyhedrin was detected by immunoblotting with a mouse monoclonal anti-polyhedrin antibody. Lane M, protein marker; lane 1, immunoprecipitate from cells co-infected with BmNPV T3 isolate and the viral stock expressing eGFP-BmLC3; lane 2, immunoprecipitate from cells co-infected with BmNPV T3 isolate and the stock expressing eGFP. Arrow indicated the polyhedrin. (**C**) Polyhedrin-eGFP co-immunoprecipitates with BmLC3. Vectors pP_ph_-Polyhedrin-eGFP and pP_ph_-eGFP were used to obtain individual viral stock. Polyhedrin-eGFP and eGFP alone were co-immunoprecipitated for SDS-PAGE and then Western blot with a rabbit polyclonal anti-BmLC3 antibody. Lane M, protein marker; lane 1, immunoprecipitate from cells infected with the viral stock expressing polyhedrin-eGFP; lane 2, immunoprecipitate from cells infected with the virus expressing eGFP. Arrow indicated BmLC3.

Classically, large toxic aggregates are enclosed selectively into the autophagosome. Autophagosomes eventually fuse with lysosomes to degrade their content. This process, where LC3 plays an important role, is termed as aggrephagy^5^. Next, colocalization of polyhedrin and the autophagosome marker BmLC3 was performed. Two viruses individually expressing fusion proteins polyhedrin-mCherry and eGFP-BmLC3 were used to co-infect BmN cells, and visualized by a confocal microscope. Some autophagosomes, ranged from 3.03–7.21 μm in diameter size, were observed in the cytoplasm. At 24 h p.i. when large number of co-infected cells displayed aggregated polyhedrin foci, polyhedrin-mCherry was not colocalized with BmLC3. It seemed that these aggregated polyhedrin-mCherry proteins were sequestered by BmLC3 (Fig. 7A, upper panel). At 48 h p.i. when aggregated polyhedrin was recovered to function, polyhedrin-mCherry was colocalized with BmLC3 to the isolation membrane of autophagosome (Fig. 7A, lower panel), suggesting that polyhedrin is involved in cellular autophagy.

These findings prompted the question of interaction between polyhedrin and BmLC3. To address it, two viruses, the wildtype BmNPV T3 isolate and a stock expressing fusion protein eGFP-BmLC3, were used for co-infection. At 48 h p.i., cells were collected, lysed and then co-immunoprecipitation was carried out with a μMACS GFP Isolation Kit. Co-purified proteins were subjected to SDS-PAGE then Western blot with a mouse monoclonal anti-polyhedrin antibody. A band with a molecular weight of approximately 28 kDa was detected (Fig. 7B). To test whether there was a direct or indirect association between polyhedrin and BmLC3, a reverse co-immunoprecipitation was performed. BmN cells were infected with a virus expressing the protein polyhedrin-eGFP. Co-purified proteins were detected with a rabbit polyclonal anti-BmLC3 antibody. A band with a molecular weight of about 13 kDa was observed (Fig. 7C). Taken together, data thus suggested strongly that polyhedrin binds directly to BmLC3.

### Cellular autophagy played a role in OB formation

**Figure 8:**
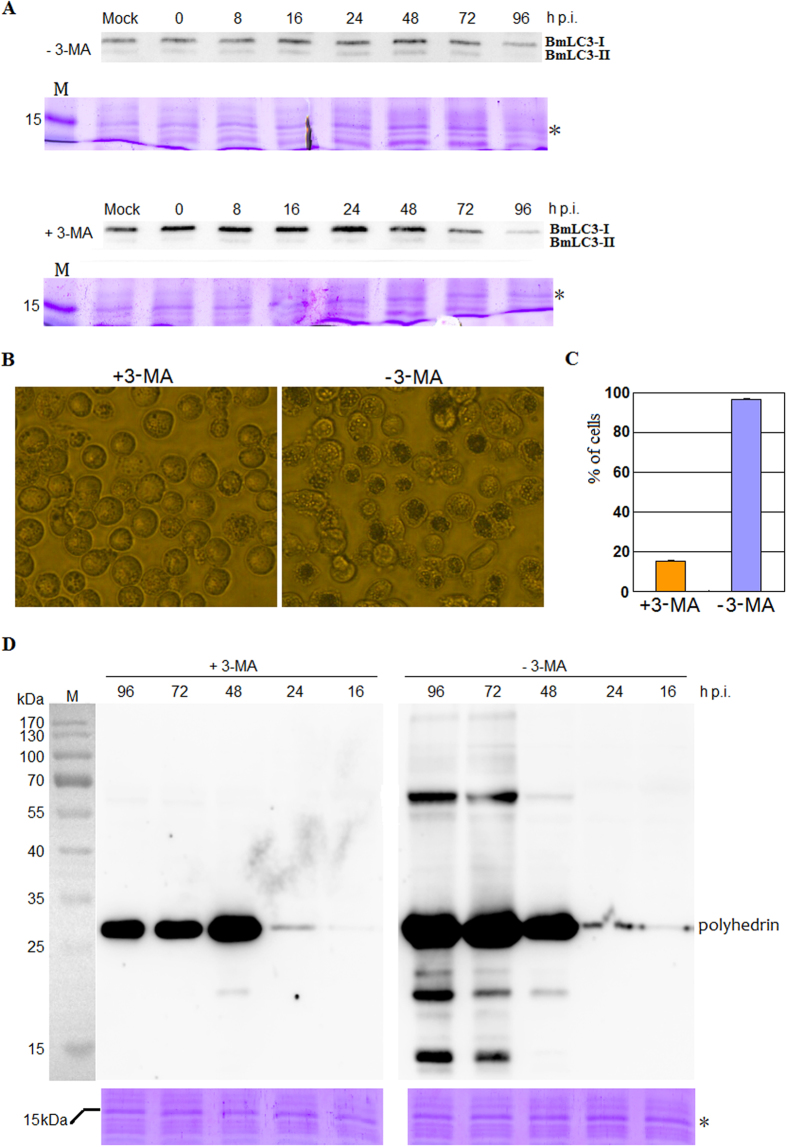
Cellular autophagy played a role in polyhedra particle production. Land M, protein marker. (**A**) Cellular autophagy was inhibited by 3-MA. (**B**) Light microscopy observations at 72 h p.i. of 3-MA-treated and untreated BmN cells infected with BmNPV T3 isolate. (**C**) Percentage of cells displaying OBs in the nucleus. Each value is calculated at 72 h p.i. from three independent infections and for each infection, 298–360 cells were scored. Error bars indicates ±SEM. (**D**) Western blot analysis of polyhedrin with a mouse monoclonal anti-polyhedrin antibody.

As described above, polyhedrin was colocalized with BmLC3 on the isolation membrane of autophagosome, prompting the question whether or not cellular autophagy played a role in polyhedra particle production. Firstly, cellular autophagy was detected during BmNPV infection. Cells were infected with the BmNPV T3 isolate, and at designated time points p.i. harvested for Western blot analysis with a rabbit polyclonal anti-BmLC3 antibody. Coomassie blue stain of total proteins was used as the internal reference in this study for normalization (Fig. 8A,D, lower panel; See also Supplementary Fig. S1). LC3 is the most widely monitored autophagy-related protein^29^. BmLC3 was detected as two bands (Fig. 8A). One is BmLC3-I which is cytosolic, and the other represents BmLC3-II that is present on isolation membranes of autophagosomes and pre-autophagosomes (Fig. 7A). During BmNPV infection, the level of BmLC3-I is stable. However the amount of BmLC3-II in 3-MA-treated cells is lower than that in untreated cells, suggesting that cellular autophagy was inhibited by 3-MA (Fig. 8A).

Supernatants were collected at 24 and 48 h p.i. for BV yield determination. No significant differences were observed in BV productions of treated and untreated infections (Data not shown). At 72 h p.i., it was found that treatment with 3-MA significantly inhibited OB production whereas a large number of OBs were observed in the nucleus of untreated cells (Fig. 8B,C). Western blot analysis also indicated that 3-MA markedly inhibited polyhedrin protein expression. From 48 to 96 h p.i., the level of polyhedrin in 3-MA-treated cells decreased, suggesting another pathway, such as ubiquitin-proteosome system, played a role in degradation of polyhedrin (Fig. 8D, left panel). Theoretically, the molecular weight of polyhedrin is 28.8 kDa. Some bands with molecular sizes larger and lower than 28.8 kDa were detected in samples from untreated BmN cells (Fig. 8D, right panel). As described above, polyhedrin colocalized with BmLC3 to the isolation membrane of autophagosome (Fig. 7A, lower panel). These bands with molecular weight lower than 28.8 kDa may represent partial degraded products of polyhedrin; and these with molecular size larger than 28.8 kDa maybe phosphorylated, polymerized and/or ubiquitinated polyhedrin proteins. Taken together, these results suggested that cellular autophagy is required for efficient production of OBs during BmNPV infection.

## Discussion

Aggregates, as toxic structures to cells, contain some misfolded and/or unfolded proteins. Once produced in the cytoplasm, aggregated proteins are refolded by some molecular chaperones and remodeling factors to obtain their active conformations thereby reducing the likelihood of protein aggregation^4^. BmNPV polyhedrin, itself was predicted with a 3D profile method to show a high fibrillation propensity, which was verified in this present study that formation of aggregates and aggresomes by polyhedrin was observed with a red fluorescent molecular rotor dye that has been used to specifically detect denatured and/or misfolded protein cargo in fixed and permeabilized cells, and then confirmed further by colocalization of polyhedrin with aggresomal markers GFP-250 and γ-tubulin. These aggregates colocalized with molecular chaperone HSC/HSP70s and recruited the mitochondria. Accompanying progress of BmNPV infection, the number of aggregates decreased. Polyhedrin is expressed at the very late stage of baculovirus life cycle in a relatively short time and a large amount, whereby folding becomes a limited step for polyhedrin active conformation. Therefore, aggregated polyhedrin needed cellular chaperones for its own protein folding process. It seemed that aggregated proteins stimulated expression of HSC/HSP70s^14^,^30^. HSC/HSP70 family chaperones, as central components of the cellular chaperone network, are frequently recruited by virus, and play a role in facilitating AcMNPV genome synthesis and release of progeny BVs^30^. It seemed that these aggregate structures may serve as “storage bins” for polyhedrin to be refolded to its active conformation with help of some chaperones. Thus, for a productive infection, it is necessary for baculovirus to form aggregates throughout its life cycle.

Moreover, cells have another important pathway in which some aggregates are delivered to the MTOC and transported to perinuclear locations to form aggresomes. To promote clearance of aggregated proteins, these structures are enwrapped to result in autophagosome which will fuse with lysosome to produce autolysosome where proteins targeted in aggresomes were degraded. Aggresomes can form in response to viral infection and play a role during infections of some viruses^7^,^8^,^9^,^10^. Aggresomes were also detected during infection of baculovirus AcMNPV by an indirect way of using anti-HSC/HSP70 and anti-ubiquitin antibodies^14^,^15^. During viral infection aggresomes are considered as sites for sequestration, inactivation and degradation of potentially harmful proteins, and play an important role for efficient progeny production. Some viruses, such as vaccinia virus, iridoviruses, and African swine fever virus employed aggresomes as “virus factories” for virus accumulation and assembly^31^. Some viruses exploit aggresomes to create a favorable host cell environment for their productive infection. For instance, the E1B-55K protein of adenovirus 5 targets the cellular Mre11-Rad50-Nbs1 complex into aggresomes to accelerate inactivation of this complexes then degradation by proteosomes, and thus aggresome formation contributes to the protection of genomic DNA for efficient viral growth^7^,^8^. Integrin α3 is also targeted to aggresomes by adenovirus E1B-55K protein and then degraded, thus may play a role in promoting release and spread of progeny virions^32^. For baculovirus AcMNPV, from 24 to 48 h p.i., aggresomes fused with lysosomes, and when their fusion with lysosomes was inhibited, expression of very late viral proteins, such as P10 and polyhedrin, was efficiently suppressed^15^, implying that aggresomes play an important role, though remained elusive, for efficient progeny polyhedra particle production. Attempts to investigate the role of baculovirus aggresomes are essential and valuable in the future.

For a long time, polyhedrin was thought to be a structural component protein to stabilize baculovirus virions released in the environment. The most striking observations in the present report were that recovered polyhedrin interacted with the autophagosome marker BmLC3 and colocalized with BmLC3 to the isolation membrane of autophagosome, implying the involvement of polyhedrin in cellular autophagy which was testified further by results that inhibition of autophagy led to remarkable decrease in polyhedrin expression and polyhedra production. Polyhedrin may function as an autophagic adapter protein to get involved in a process of selective autophagy through a so-called LIR (LC3-interacting region) motif though it should be confirmed further. As an autophagic adapter, three features, a direct interaction between the autophagic adapter and the LC3, the inherent ability to polymerize or aggregate as well as to specifically recognize substrates, are required^33^. The involvement of an adapter in cellular selective autophagy is always regulated by post-translational modifications such as phosphorylation and ubiquitination^34^,^35^. Besides polyhedrin protein with the theoretical molecular weight of 28.8 kDa was detected, some bands with molecular size larger than 28.8 kDa were observed. These bands may represent polyhedrin modified post-translationally. Some bands that may be partial degraded products of polyhedrin with molecular weight lower than 28.8 kDa, were also found. Clearly, the involvement of polyhedrin in autophagy to regulate production of polyhedra particles needs to be clarified.

In conclusion, the results presented here provide novel insight into the role of baculovirus polyhedrin. In addition to formation of polyhedra in the nucleus of infected cells, highly expressed polyhedrin is involved in the aggresome-autophagy pathway in the cytoplasm, then to regulate the production of polyhedra.

## Methods

### Cells, virus, primers and antibodies

The *B. mori* cell line BmN was cultured at 27 °C in TC-100 insect medium (AppliChem, Darmstadt, Germany) supplemented with 10% (v/v) heat-inactivated fetal bovine serum (Gibco-BRL, Life technologies, Newcastle, Australia), 100 μg/ml of penicillin, and 30 μg/ml of streptomycin. The BmNPV T3 isolate^36^ was propagated in BmN cells. Virus titers were determined by end-point dilution and were expressed as TCID_50_/ml, as described previously^37^. BmN cells were infected with BmNPV BVs at an MOI of 10 TCID_50_/cell.

The *E. coli* strain BmDH10Bac was provided by Dr. Enoch Y. Park of the Department of Applied Biological Chemistry, Shizuoka University, Japan^38^. The aggresomal marker GFP-250 expression vector was gift from Prof. Elizabeth S. Sztul of University of Alabama^24^. Primers used were shown in supplementary Table S1.

Antibodies, such as rabbit polyclonal antibody to HSC/HSP70 (sc-33575, Santa Cruz Biotechnology Inc., Santa Cruz, CA, USA), mouse monoclonal anti-ubiquitinylated protein antibody clone FK2 (04–263, Millipore Corporation, Billerica, MA, USA), rabbit polyclonal antibody to γ-tubulin (T5192, Sigma-Aldrich, Inc., St. Louis, USA), mouse monoclonal antibody to BmNPV polyhedrin (A gift from Prof. Wen-Bin Wang of Jiangsu University, China), rabbit polyclonal antibody^39^ to BmLC3 (SilkDB: BGIBMGA011783-PA) (A gift provided by Prof. Yang Cao of South China Agricultural University, China) were used.

### ProteoStat staining assay

A ProteoStat^®^ aggresome detection kit (Enzo life sciences Inc., Plymouth Meeting, PA, USA) was used to characterize the aggregates and aggresomes in infected BmN cells. All components of this kit were prepared according to the manufacture’s instructions. Monolayer of cells was grown on cell culture dishes (35.0 mm in diameter) (Corning Incorporated, Corning, NY, USA) and either mock- or infected with BmNPV BVs. After 1 h for infection, fresh medium was added after three washes with serum-free medium. This time point was defined as 0 h p.i. At various times p.i., cells were washed with 1 × PBS and fixed with 4% paraformaldehyde for 30 min at room temperature. After washed with excess 1 × PBS, cells were permeabilized with 0.5% Triton X-100, 3 mM EDTA in 1 × PBS for 30 min. The cells were again washed twice with 1 × PBS and the ProteoStat dye was added at a 1:2000 dilution for 30 min at room temperature. Images were acquired with a Leica TCS SP5 confocal laser-scanning microscope.

### Donor plasmids

Plasmid pFastBac^TM^1 was purchased from Invitrogen Life technologies (Carlsbad, CA, USA). The vector pFast was constructed previously^40^. Details on construction of all donor plasmids used in this work were available in supplementary Table S2. The identities of all plasmid constructs were verified by DNA sequencing and/or restriction digestion.

### Transposition and cell transfection

Transposition of eGFP, mCherry and related constructs listed in supplementary Table S2 into BmNPV genome in *E. coli* strain BmDH10Bac containing the BmNPV bacmid and a helper plasmid pMON7124, transfection of positive bacmids were performed as previously^41^. BV stocks obtained from transfections were used for infection. The P2 viral stocks were used for fluorescent cell visualization under a Leica TCS SP5 confocal laser scanning microscope.

### Immunofluorescence microscopy

BmN cells were either mock- or infected with BVs. At various times p.i., cells were fixed and permeabilized using the above protocol, incubated in 1 × PBS containing 3% bovine serum albumin, reacted with primary antibody diluted adequately in 1 × PBS for 1 h at room temperature, washed in excess 1 × PBS, and then reacted with fluorescein-labeled secondary antibody for 1 h at room temperature. Stained cells were visualized using a Leica TCS SP5 confocal laser scanning microscope for fluorescence detection.

### Nocodazole treatment

BmN cells were kept in TC-100 medium containing 20 μM nocodazole dissolved in dimethyl sulfoxide (DMSO) after 1 h of virus inoculation. Untreated cells were maintained in medium containing the same concentration of DMSO.

### Treatment of cells with 3-MA

Monolayer of BmN cells were grown in a 25-cm^2^ flask and infected with BVs at an MOI of 10 TCID_50_/cell in the presence of 3-MA to a final concentration of 12.5 mM. After 1 h of inoculation, cells were kept in 4 ml fresh medium containing 3-MA after three washes with serum-free medium. At selected time points p.i., a small amount of culture medium was collected and BV production was determined by end point dilution^37^. Infected cells were harvested for Western blot analysis.

### Co-immunoprecipitation

Infected BmN cells were harvested at 48 h p.i., and lysed in buffer (25 mM Tris-HCl pH7.4, 150 mM NaCl, 1 mM EDTA, 1% v/v NP-40, 5% v/v glycerol) with Complete, EDTA-free protease and PhosSTOP phosphatase inhibitor mixture (Roche Applied Science). Co-immunoprecipitations were performed using a μMACS GFP Isolation Kit (Miltenyi Biotec GmbH, Bergisch Gladbach, Germany) according to the manufacturer’s instructions. The obtained immunoprecipitates were used for Western blot analysis.

### Prediction of aggregation-prone regions of BmNPV polyhedrin

The prediction of aggregation-prone profiles within polyhedrin amino acid sequence was performed using a 3D profile method in the ZipperDB database^42^.

## Additional Information

**How to cite this article**: Guo, Z.-J. *et al.* Characterization of aggregate/aggresome structures formed by polyhedrin of *Bombyx mori* nucleopolyhedrovirus. *Sci. Rep.* **5**, 14601; doi: 10.1038/srep14601 (2015).

## Supplementary Material

Supplementary Dataset 1
