# Supplementary material for: Characterization of aggregate/aggresome structures formed by polyhedrin of Bombyx mori nucleopolyhedrovirus
Source: Sci Rep. 2015 Oct 6;5:14601. doi: 10.1038/srep14601 (PMC4594129; doi:10.1038/srep14601)
Supplement: Supplementary Dataset 1 [file srep14601-s1.doc]

**Characterization of aggregate/aggresome structures formed by polyhedrin of *Bombyx mori* nucleopolyhedrovirus**

Zhong-Jian Guo^1^, Liu-Xing Tao^1^, Xian-Yun Dong^1^, Meng-Han Yu^1^, Ting Tian^1^ & Xu-Dong Tang^2^

^1^ Institute of Life Sciences, Jiangsu University, 301# Xuefu Road, Zhenjiang 212013, Jiangsu, P.R. China

^2^ College of Biotechnology, Jiangsu University of Science and Technology, 2# Mengxi Road, Zhenjiang 212018, Jiangsu, P.R. China

Correspondence and requests for materials should be addressed to Z-J. G. (email: [gzh762677@ujs.edu.cn](mailto:gzh762677@ujs.edu.cn)) or X-D. T. (email: [xudongt@aliyun.com](mailto:xudongt@aliyun.com))

**Supplementary data**

| Supplementary Table S1. Primers used in this study. | |
| --- | --- |
| Primer name | Primer sequence^†^(5'→3') |
| Pie1-F | GCGGTCCGATCAATGTCTTTGTGATGCGCG |
| Pie1-R | AGAATTCCGCGCGCTTGGATCCTTGGTTGTTCACGATCGTGTCG |
| GFP-F | GCGCGGGATCCATGGTGAGCAAGGGCGAGGAG |
| GFP-R | GCAAGAAGCTTTCACTTGTACAGCTCGTCCATGC |
| GFP(*Eco*RI-Linker)-R | GCGCGGAATTC***TGAACCACCACCTGA***CTTGTACAGCTCGTCCATGC |
| GFP(*XhoI*-Linker)-F | GCAAGCTCGAGGCATGCGGTACC***TCAGGTGGTGGTTCA***ATGGTGAGCAAGGGCGAGGAG |
| GFP-250-R | GCGCGAAGCTTCCTACGTTGAATATAAGAGCC |
| Polyhedrin-F | CTTCCGAATTCATGCCGAATTATTCATACAC |
| Polyhedrin(TAA-Null)-R | CTTCCCTCGAGATACGCCGGACCAGTGAACAG |
| BmLC3-F | CGAATTCATGAAATTCCAATACAAAGAAG |
| BmLC3-R | TCTCGAGTTAATTTCCATAGACATTTTCG |

^†^Restriction sites are underlined. Italic and bold sequence encodes a flexible peptide linker SGGGS^1^.

**Reference**

1. Morell, M., Espargaró, A., Avilés, F.X. & [Ventura](http://scholar.google.com/citations?user=K7M0_kgAAAAJ&hl=zh-CN&oi=sra), S. [Detection of transient protein–protein interactions by bimolecular fluorescence complementation: the Abl-SH3 case](http://onlinelibrary.wiley.com/doi/10.1002/pmic.200600966/full). *Proteomics* **7**, 1023–1036 (2007).

| Supplementary Table S2. Constructions of donor plasmids used in this work. | |
| --- | --- |
| Vectors | Construction |
| pP_ie1_ | A *Cpo*I-*Eco*RI fragment containing the *ie1* promoter was amplified with primers Pie1-F and Pie1-R using BmNPV T3 isolate genomic DNA as template and was inserted into *Cpo*I-*Eco*RI-digested pFast, to produce plasmid pP_ie1_. |
| pP_ie1_-eGFP | The enhanced green fluorescence protein (eGFP) encoding sequence with *Bam*HI/*Hin*dIII sites was amplified with primers GFP-F and GFP-R using the plasmid pEGFP-C1 as a template, then inserted into *Bam*HI/*Hin*dIII-digested pP_ie1_, to result in pP_ie1_-eGFP. |
| pP_ie1_-GFP-250 | The fragment harboring aggresomal marker GFP-250 encoding region was PCR amplified with primer pair GFP-F/GFP-250-R, and introduced into corresponding restriction sites of pP_ie1_ to yield plasmid pP_ie1_-GFP-250. |
| pP_ie1_-eGFP(*EcoR*I-Linker) | The eGFP encoding sequence was PCR amplified with primers GFP-F and GFP(*Eco*RI-Linker)-R, then cloned into *Bam*HI-*Eco*RI-digested pP_ie1_. |
| pP_ie1_-eGFP-BmLC3 | The silkworm BmLC3 (SilkDB: [BGIBMGA011783-PA](http://silkworm.swu.edu.cn/cgi-bin/gbrowse/silkdb/?name=BGIBMGA011783-TA)) encoding fragment was RT-PCR amplified with primer pair BmLC3-F/BmLC3-R. This sequence was introduced into *Eco*RI-*Xho*I sites of p pP_ie1_-eGFP(*EcoR*I-Linker) to express an eGFP-tagged autophagosome marker, BmLC3^1,2^. |
| pP_ph_-eGFP | The eGFP encoding sequence retrieved from pP_ie1_-eGFP was inserted into corresponding sites of pFastBac^TM^1. |
| pP_ph_-mCherry | The mCherry encoding sequence was amplified with primers GFP-F and GFP-R, and plasmid pLVX-mCherry-N1 as a template, and then cloned into *Bam*HI/*Hin*dIII sites of pFastBac^TM^1. |
| pP_ph_-mCherry(*Xho*I-Linker) | The mCherry encoding sequence was amplified with primer pair GFP(*Xho*I-Linker)-F/GFP-R and vector pLVX-mCherry-N1 as a template. This fragment was inserted into *Xho*I-*Hin*dIII-digested pFastBac^TM^1 to obtain plasmid pP_ph_-mCherry(*Xho*I-Linker). |
| pP_ph_-Polyhedrin-mCherry | The BmNPV *polyhedrin* gene was amplified with primers Polyhedrin-F and Polyhedrin(TAA-Null)-R using BmNPV T3 isolate genomic DNA as template then was introduced into *Bam*HI-*Xho*I-digested pP_ph_-mCherry(*Xho*I-Linker), to produce plasmid pP_ph_-Polyhedrin-mCherry. |
| pP_ph_-Polyhedrin-eGFP | Construction of pP_ph_-Bmpoly-eGFP is carried out according to the procedure described above. Primer pair GFP(*XhoI*-Linker)-F/GFP-R and vector pEGFP-C1 as a template were used. The eGFP encoding sequence was cloned into pFastBac^TM^1 to result in plasmid pP_ph_-eGFP(*Xho*I-Linker), then the *polyhedrin* was inserted into pP_ph_-eGFP(*Xho*I-Linker) to produce donor vector pP_ph_-Polyhedrin-eGFP. |

**References**

1. Franzetti, E. *et al*. Autophagy precedes apoptosis during the remodeling of silkworm larval midgut. *Apoptosis* **17**, 305–324 (2013).
2. Tian, L. *et al*. 20-hydroxyecdysone upregulates *Atg* genes to induce autophagy in the Bombyx fat body. *Autophagy* **9**, 1172–1187 (2013).

Supplementary figure.


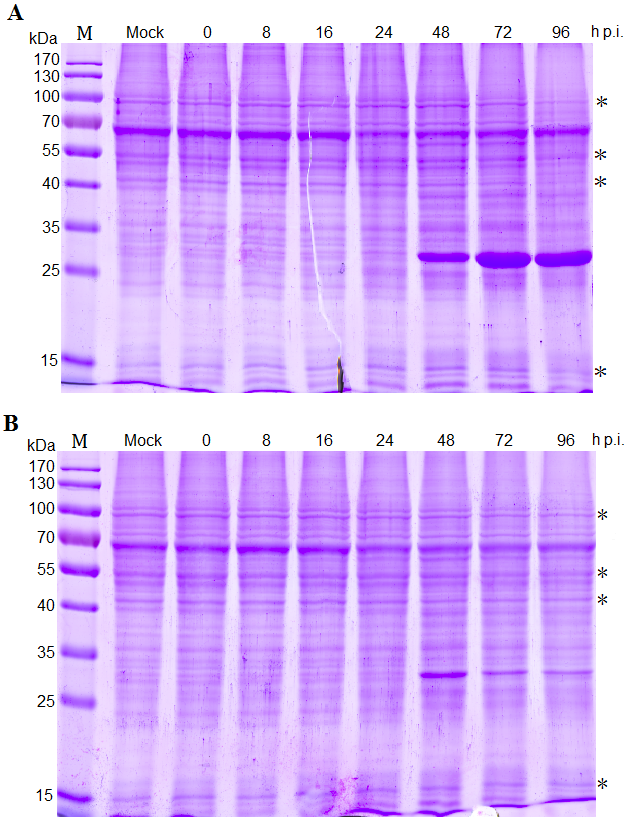


Figure. S1. Coomassie blue stain of total proteins adopted as internal reference. For that the most commonly used housekeeping reference β-actin was degraded during BmNPV infection^1^, Coomassie blue stain of total proteins, which was adopted previously^2^, was used as the internal reference in this study for normalization. Total proteins from untreated (A) and 3-MA-treated (B) cells collected at time points p.i. were subjected to SDS-PAGE and Coomassie blue staining. These * symbols suggested potential of these bands as references.

**References**

1. Katsuma, S., Tsuchida, A., Matsuda-Imai, N., Kang, W. & Shimada, T. Role of the ubiquitin–proteasome system in *Bombyx mori* nucleopolyhedrovirus infection. *J. Gen. Virol.* **92**, 699–705 (2011).
2. Gorovits, R., Moshe, A., Ghanim, M. & Czosnek, H. Recruitment of the host plant heat shock protein 70 by tomato yellow leaf curl virus coat protein is required for virus infection. *PLoS ONE* **8**, e70280 (2013).
